# The Efficiency and Mechanism of FeOCl/Ce-Catalyzed Persulfate for the Degradation of Caffeine Under Visible Light

**DOI:** 10.3390/molecules30224381

**Published:** 2025-11-13

**Authors:** Zhao Bai, Mingyue Hu, Minrui Li, Weidong Wu, Chi Zhou, Yuru Wang

**Affiliations:** 1 Shaanxi Key Laboratory of Environmental Monitoring and Forewarning of Trace Pollutions, Shaanxi Provincial Environmental Monitoring Central Station, Xi’an 710006, China; sgf369@126.com (Z.B.); zhou04280428@163.com (C.Z.); 2 Department of Environmental Science, School of Geography and Tourism, Shaanxi Normal University, Xi’an 710119, China; 15607122889@163.com (M.H.); liminrui@snnu.edu.cn (M.L.)

**Keywords:** FeOCl/Ce composite, rare-earth doping, persulfate, hydroxyl radical, caffeine degradation

## Abstract

Despite extensive work on FeOCl-based photocatalysts, few studies have explored rare-earth (Ce) doping to simultaneously tune bandgap, suppress charge recombination, and enhance visible light-driven persulfate (PS) activation for the degradation of emerging contaminants. This study synthesized FeOCl/Ce composite photocatalysts via a partial pyrolysis method and systematically characterized their physicochemical properties. The results show that Ce doping significantly lowers the bandgap energy of the photocatalyst, enhances its visible light absorption ability, and effectively suppresses the recombination of photogenerated electron–hole pairs, thereby markedly improving photocatalytic performance under visible light. Analyses including XRD, EDS, XPS, and FT-IR confirm that Ce is incorporated into the FeOCl matrix and modulates the radial growth behavior of FeOCl without altering its intrinsic crystal structure. Morphological observations reveal that FeOCl/Ce exhibits a uniform nanosheet layered structure, with larger particles formed by the aggregation of smaller nanosheets. The nitrogen adsorption–desorption isotherm of FeOCl/Ce shows characteristics of Type IV with a relatively small BET surface area. The broadened optical absorption edge of FeOCl/Ce and the results of PL spectra and I-T curves further confirm its enhanced visible light absorption capacity and reduced electron–hole recombination compared to pure FeOCl. At an initial caffeine (CAF) concentration of 10 μM, FeOCl/Ce dose of 0.5 g/L, PS concentration of 1 mM, and initial pH of 5.06, the FeOCl/Ce-catalyzed PS system under visible light irradiation can degrade 91.2% of CAF within 30 min. An acidic environment is more favorable for CAF degradation, while the presence of SO_4_^2−^, Cl^−^, and NO_3_^−^ inhibits the process performance to varying degrees, possibly due to competitive adsorption on the photocatalyst surface or quenching of reactive species. Cyclic stability tests show that FeOCl/Ce maintains good catalytic performance over multiple runs. Mechanistic analysis indicates that ^•^OH and holes are the dominant reactive species for CAF degradation, while PS mainly acts as an electron acceptor to suppress electron–hole recombination. Overall, the FeOCl/Ce photocatalytic system demonstrates high efficiency, good stability, and visible light responsiveness in CAF degradation, with potential applications for removing CAF and other emerging organic pollutants from aquatic environments.

## 1. Introduction

Pharmaceuticals and personal care products (PPCPs) comprise a wide range of compounds used to prevent or treat diseases in humans and animals, as well as personal care products intended to improve daily life. They include thousands of chemicals such as antibiotics, hormones, lipid regulators, analgesics, UV filters, disinfectants, and cosmetics [1]. Most PPCPs are highly polar and have low volatility, which makes them readily migrate into aquatic environments, particularly through sources such as domestic sewage, medical wastewater, and aquaculture wastewater [2,3]. Although PPCPs typically occur in the environment at low concentrations (ng/L–μg/L) [4], they can cause serious ecotoxicological effects and pose substantial threats to organisms and ecosystems [2,5]. Many PPCPs can persist in water and accumulate in organisms, potentially disrupting endocrine systems or promoting antimicrobial resistance, thereby exacerbating ecosystem imbalance and causing irreversible impacts on ecosystems and human health [6]. Conventional wastewater treatment plants lack dedicated removal technologies for PPCPs, making treated effluent an important source of PPCPs in the environment [7,8]. Therefore, there is an urgent need to develop advanced treatment technologies to efficiently degrade PPCPs.

Among the water treatment technologies, advanced oxidation processes (AOPs) are highly promising for removing a wide range of pollutants. AOPs employ highly reactive radical species, such as hydroxyl radicals (^•^OH), sulfate radicals (SO_4_^•−^), and superoxide radicals (O_2_^•−^) as primary oxidants to degrade recalcitrant organic contaminants and ultimately mineralize them into CO_2_, H_2_O, and inorganic ions [9]. As a type of AOPs, semiconductor photocatalytic advanced oxidation technology has attracted extensive attention in recent years [10,11]. The energy band structure of a semiconductor photocatalyst consists of a valence band (VB) filled with electrons and an empty conduction band (CB), separated by a bandgap Eg. When the photon energy (hν) exceeds Eg, electrons are excited from the VB to the CB, producing photogenerated electrons in the CB and holes (h^+^) in the VB. The photogenerated electrons can reduce surface-adsorbed oxygen to form O_2_^•−^, while photogenerated holes oxidize water molecules to form ^•^OH. The resulting O_2_^•−^, ^•^OH, and *h^+^* are powerful oxidants capable of decomposing recalcitrant organic pollutants into simple inorganic substances [12].

Among semiconductor photocatalysts, iron oxychloride (FeOCl) has become a recent research focus due to its unique advantages [13]. The iron in FeOCl exists as a mixture of Fe(II) and Fe(III), and the distinctive structure of FeOCl facilitates internal charge transfer that enables interconversion between Fe(II) and Fe(III) [14]. FeOCl has a relatively narrow bandgap, allowing an effective response to photons in the visible light region [15]. Moreover, the stable layered Fe–O–Cl arrangement in the FeOCl crystal structure endows it with both good chemical stability and distinctive catalytic active sites. However, pure-phase FeOCl suffers from rapid recombination of photogenerated charge carriers during photocatalytic processes, which negatively affects its performance [16].

To address this issue, doping with rare-earth elements is an effective approach to improve the photocatalytic performance of semiconductors. The 4f orbitals of rare-earth elements exhibit multiple levels of splittings and multilevel transition pathways, allowing rare-earth ions (such as La^3+^, Ce^3+^/Ce^4+^, Nd^3+^, etc.) to act as electron-trapping wells during photocatalysis [17]. By accepting photogenerated electrons and undergoing valence cycling (e.g., Ce^3+^ → Ce^4+^ → Ce^3+^), they prolong the separation lifetime of electron–hole pairs and thus reduce the recombination rate. At the same time, differences between the ionic radii of rare-earth ions and those of the semiconductor lattice ions make it easy for doping to induce oxygen vacancies or lattice defects on the catalyst surface or within the lattice [18,19]. These defect sites can not only enhance the adsorption capacity for pollutants but also serve as new catalytically active centers, further accelerating photocatalytic reaction rates. Based on these characteristics, rare-earth-doped photocatalysts are regarded as an important direction for developing highly active photocatalytic materials. However, the exact contribution of doped rare-earth ions to the enhancement of photocatalytic activity still requires further investigation.

In addition, introducing external electron acceptors has been proven to be another effective strategy for optimizing photocatalytic systems. Among these, hydrogen peroxide (H_2_O_2_), peroxymonosulfate (PMS, e.g., KHSO_5_), and persulfate (PS, e.g., Na_2_S_2_O_8_) are the most commonly used electron scavengers [14,20,21,22]. On the one hand, they can rapidly capture photogenerated electrons, directly suppressing electron–hole recombination. On the other hand, the electron-capture process produces extra reactive oxygen species (e.g., ^•^OH and SO_4_^•−^), which significantly enhance the system’s oxidative degradation capability. It is worth noting that, compared with·OH (*E_0_* = 1.8–2.7 V), the sulfate radical (SO_4_^•−^) has a higher redox potential (*E_0_* = 2.5–3.1 V) and exhibits greater stability and selectivity under neutral to alkaline conditions [15]. Therefore, photocatalytic systems supplemented with persulfates (PMS/PS) often show superior performance in wastewater treatment.

Against the above background, this study selected the rare-earth element Ce to dope FeOCl and investigated the performance and reaction mechanism of FeOCl/Ce photocatalysts in activating persulfate (PS) for the degradation of target PPCPs under visible light.

To the best of our knowledge, the efficiency and mechanism of visible light-driven PS activation by FeOCl/Ce catalyst have not been explored. A series of characterization techniques was used to examine the structure, morphology, properties, and chemical states of the elements in FeOCl/Ce. Caffeine (CAF, molecular formula: C_8_H_10_N_4_O_2_; molecular weight: 194.19), a component widely present in food and pharmaceuticals, was selected as a representative of PPCPs due to its frequent occurrence in aqueous environments and recalcitrant nature to biodegradation [23]. The performance of FeOCl/Ce in activating PS for CAF removal in aqueous solution was evaluated under visible irradiation. The catalytic degradation efficiency of FeOCl/Ce in activating PS in complex water bodies was examined by varying different parameters of the system, including photocatalyst dose, PS concentration, solution pH, and addition of common anions in water. The stability and reusability of FeOCl/Ce were explored through recycling experiments and soluble iron leaching experiments in the system. Radical quenching experiments were used to identify the reactive species in the system. The results of this study will provide theoretical guidance for the practical application of FeOCl-based photocatalysts.

## 2. Results and Discussion

### 2.1. Characterization of the Structure and Properties of FeOCl/Ce

**Figure 1:**
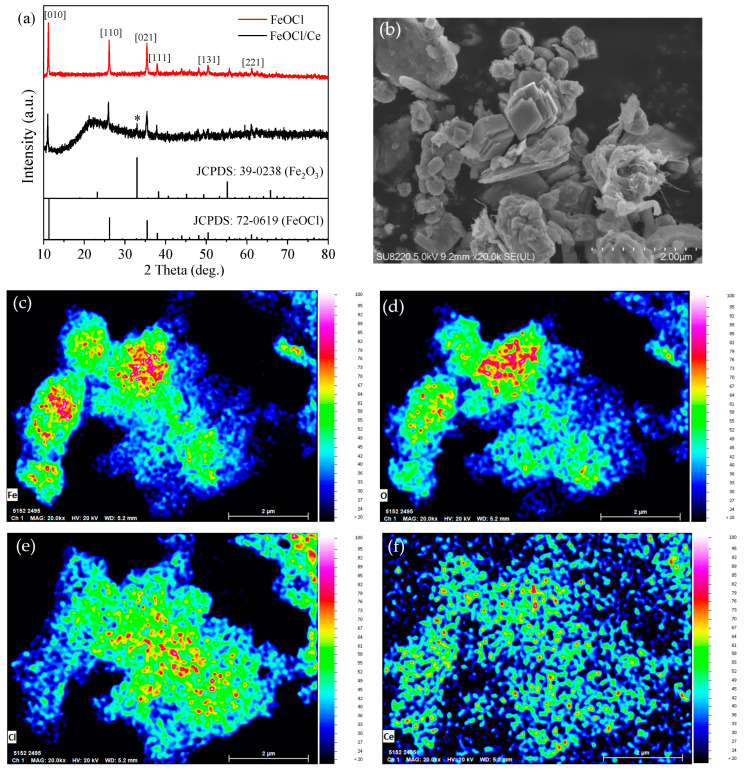
(**a**) XRD patterns of FeOCl and FeOCl/Ce (* indicates the peak of Fe_2_O_3_ at 2*θ* = 32.7°), (**b**) SEM image of FeOCl/Ce, and element mapping images of (**c**) Fe, (**d**) O, (**e**) Cl, and (**f**) Ce in FeOCl/Ce.

XRD was employed to characterize the crystal structure and phase composition of FeOCl/Ce, and the results were compared with pure FeOCl (Figure 1a). No fundamental alterations in the lattice planes or interplanar spacing of FeOCl were observed upon Ce incorporation, as evidenced by the unchanged positions of major diffraction peaks. This phenomenon may arise from the amorphous state of Ce within FeOCl or its low doping concentration [24]. Notably, the intensity of the diffraction peaks of FeOCl/Ce was reduced, and fluctuations appeared at 2*θ* = 21.1°, which could be attributed to the effect of Ce incorporation on the radial growth of FeOCl crystallites. Additionally, the XRD pattern of FeOCl/Ce revealed an impurity peak corresponding to Fe_2_O_3_ at 2*θ* = 32.7°, presumably formed during the heating step of the preparation process.

In order to observe the morphology, microstructure, and elemental composition of FeOCl/Ce more directly, SEM-EDS characterization was performed (Figure 1b–f). It can be seen that FeOCl/Ce exhibits a uniform nanosheet structure with layered stacking and slight agglomeration. Similar to FeOCl as observed by previous studies, some of the nanosheets can self-assemble into three-dimensional flower-like structures to lower the surface energy [25]. Larger particles are formed by the aggregation of smaller nanosheets. Overall, the synthesized catalyst exhibits loose packing, which is beneficial for the exposure of active sites and the uniform dispersion of pollutants, thereby enhancing photocatalytic activity. The EDS spectra (Figure 1c–f) reveal the elemental distribution of the photocatalyst. It can be seen that Fe, O, Cl, and Ce are uniformly distributed in FeOCl/Ce, confirming that Ce has been successfully incorporated into FeOCl. Table 1 presents the surface elemental composition of FeOCl/Ce. Surface elemental analysis enables a semi-quantitative determination of the relative percentage of constituent elements in the photocatalyst. Results confirm the presence of Ce in the synthesized catalyst with an extremely low relative content, verifying successful trace doping of Ce. The presence of Al is due to the detection of the substrate during the testing process [26].

**Figure 2:**
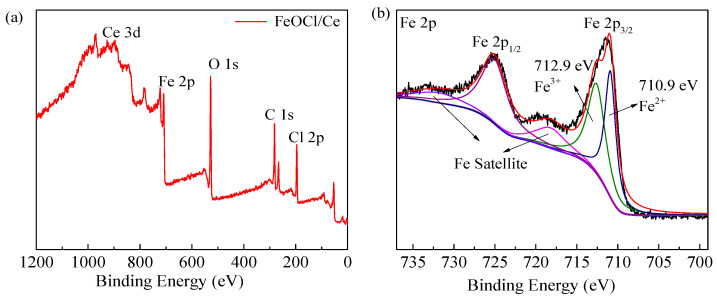

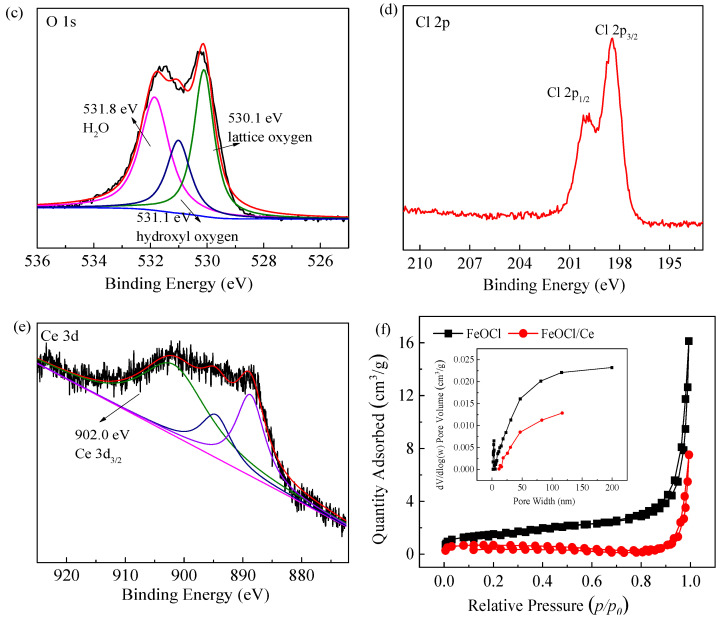
XPS analysis of FeOCl/Ce: (**a**) total scan, (**b**) Fe 2p, (**c**) O 1s, (**d**) Cl 2p, (**e**) Ce 3d, and N_2_ adsorption–desorption isotherms curves of FeOCl and FeOCl/Ce (**f**) (the inset showing the pore-size distribution).

Given that photocatalytic reactions occur at the surface of photocatalysts, investigating surface properties of photocatalysts is crucial. Therefore, XPS was utilized to analyze the types, characteristics, and valence states of surface elements in the FeOCl/Ce (Figure 2). The XPS spectra were calibrated using the C 1s peak at a binding energy of 284.6 eV. As shown in Figure 2a, the catalyst contains Fe, O, Cl, and Ce, further confirming that Ce was successfully doped into FeOCl. In Figure 2b, the Fe 2p spectrum exhibits two main peaks, corresponding to Fe 2p_1/2_ and Fe 2p_3/2_. High-resolution fitting of Fe 2p_3_/_2_ reveals two characteristic peaks at 712.9 eV and 710.9 eV, corresponding to Fe^3+^ and Fe^2+^, respectively, indicating the coexistence of Fe in +2 and +3 oxidation states. In Figure 2c, the O 1s peak is fitted to three peaks. The peak at a binding energy of 531.8 eV corresponds to H_2_O molecules adsorbed on the surface, the peak at 531.1 eV corresponds to hydroxyl oxygen, and the peak at 530.1 eV corresponds to lattice oxygen. The peaks at 199.9 eV and 198.4 eV in Figure 2d correspond to Cl 2p_1/2_ and Cl 2p_3/2_, respectively, confirming the presence of Cl^−^. In Figure 2e, a characteristic peak at a binding energy of 902.0 eV is observed, assigned to Ce 3d_2/3_, verifying the existence of Ce^3+^ [27].

Figure 2f shows the N_2_ adsorption–desorption isotherm of the synthesized catalyst (with an inset illustrating the pore size distribution), which exhibits the characteristics of a type IV isotherm with an H3-type hysteresis loop. The pore size distribution reveals that the FeOCl/Ce catalyst has both mesoporous and macroporous structures. The BET specific surface area of FeOCl/Ce is 2.04 m^2^/g, lower than that of pure FeOCl (5.40 m^2^/g), indicating a further reduction upon Ce incorporation. This contributes to the weak dark adsorption capacity of FeOCl/Ce for the target contaminant. Nevertheless, reactive species in the system are the primary factors governing target compound degradation.

**Figure 3:**
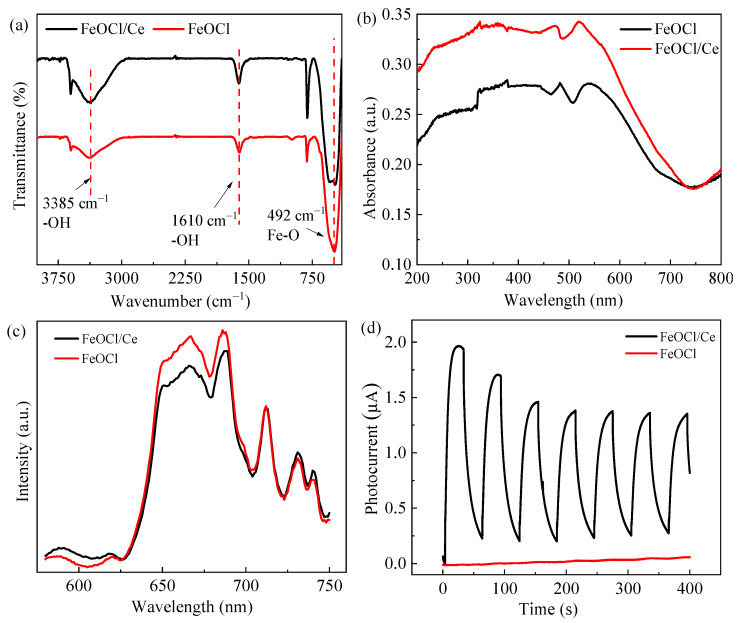
(**a**) FT-IR spectrum, (**b**) UV-Vis diffuse reflectance spectra, (**c**) PL spectra of FeOCl and FeOCl/Ce, and (**d**) transient photocurrent responses (I-T curves).

Figure 3a compares the FT-IR spectra of FeOCl and FeOCl/Ce. The spectra of the synthesized catalysts exhibit absorption peaks at wavenumbers of 3385, 1610, and 492 cm^−1^. The absorption peaks at 3385 and 1610 cm^−1^ correspond to the stretching and bending vibrations of the –OH groups, respectively, which are due to H_2_O molecules adhering to the catalyst surface, while the absorption peak at 492 cm^−1^ is characteristic of the Fe–O stretching vibrations [28]. In the wavenumber range of 400–750 cm^−1^, FeOCl/Ce displays two distinct absorption peaks, whereas FeOCl exhibits only one peak, suggesting that the incorporation of Ce induces structural modifications in the infrared spectroscopic profile of FeOCl. Notably, no new peaks appear in the FT-IR spectrum of FeOCl/Ce, which may indicate that the doping of Ce is at a trace level. The UV-visible diffuse reflectance spectra of FeOCl and FeOCl/Ce are illustrated in Figure 3b. The spectra indicate that both FeOCl and FeOCl/Ce exhibit good light absorption capability across the visible light range, demonstrating the excellent visible light responsiveness of the synthesized photocatalysts. It can also be observed that doping with Ce expands the light absorption edge of FeOCl, increasing the utilization of visible light. Therefore, FeOCl/Ce can be potentially promoted for practical applications.

Photoluminescence (PL) spectra are significant for evaluating the photocatalytic performance of catalysts. Generally, a lower intensity of specific peaks in the PL spectrum indicates a reduced recombination rate of photogenerated charge carriers [29]. The PL spectra of the two samples (Figure 3c) show multiple sub-bandgap emissions characteristic peaks in the visible region, which may arise from electronic transitions between the valence and conduction bands, or between excitonic energy bands and the valence band. Comparative analysis reveals that the characteristic peaks of FeOCl/Ce beyond 700 nm overlap with those of FeOCl, whereas its peaks in the range of 650–700 nm are significantly lower than those of FeOCl. Peak 1 (near 667 nm) is likely attributed to band-to-band recombination (i.e., electron transition from the conduction band to the valence band), while peak 2 (near 688 nm) might be associated with surface defect-induced recombination (e.g., oxygen vacancies). Both samples exhibited similar intensity for peaks near 712 nm and 731 nm, indicating that deep-level trap states (e.g., bulk defects) are not altered by Ce doping. Thus, it can be inferred that Ce doping predominantly suppresses the band-to-band and surface defect-induced recombination of photogenerated charge carriers, thereby enhancing its photocatalytic activity.

Photoelectrochemical measurements provide direct insights into the migration, separation, and recombination of photogenerated electrons and holes at the surface of photocatalysts [30]. As shown in Figure 3d, under identical experimental conditions, the I-T curves of FeOCl and FeOCl/Ce electrode systems were recorded over six on-off cycles of a xenon lamp. Upon lamp activation, the system instantly generates a photocurrent, which diminishes significantly when the lamp is turned off. Notably, the photocurrent intensity of FeOCl/Ce is substantially higher than that of pure FeOCl, indicating that Ce doping markedly enhances the photocurrent response of FeOCl.

Furthermore, the mechanism of photocurrent generation involves the diffusion of photogenerated electrons toward the surface of the working electrode, whereas photogenerated holes react with acceptors in the test electrolyte solution and are thereby consumed [29]. Thus, Ce doping facilitates the migration and separation of photogenerated electron-hole pairs, which in turn enhances photocatalytic activity.

### 2.2. FeOCl/Ce-Catalyzed PS for the Degradation of CAF Under Visible Light

#### 2.2.1. Evaluation of the Catalytic Performance of FeOCl/Ce

**Figure 4:**
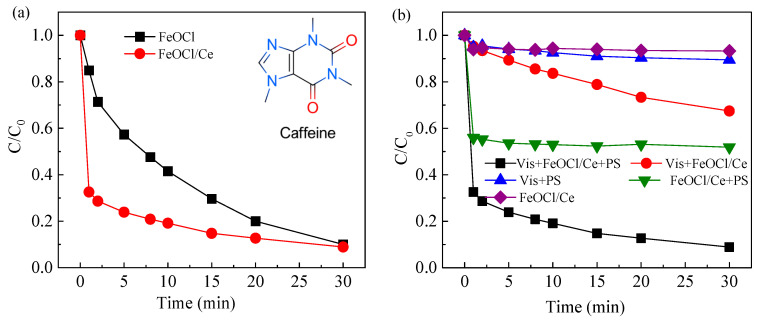
(**a**) Performance of FeOCl and FeOCl/Ce for activating PS under visible light to degrade CAF; (**b**) performance of FeOCl/Ce on the degradation of CAF in different systems. Experimental conditions: [CAF] = 10 μM, [PS] = 1 mM, [catalyst] = 0.5 g/L, [initial pH] = 5.06.

The catalytic efficiency of FeOCl/Ce was evaluated via CAF degradation under the following conditions: initial CAF concentration of 10 μM, photocatalyst dosage of 0.5 g/L, PS concentration of 1 mM, and initial system pH of 5.06. Figure 4a compares the efficacy of FeOCl and FeOCl/Ce in activating PS under visible light for the degradation of CAF, revealing that both materials achieve over 90% CAF removal within 30 min. Notably, at 1 min of reaction, the CAF degradation rate by FeOCl/Ce reaches 67.5%, significantly higher than that by FeOCl (16.1%). UV visible diffuse reflectance spectroscopy (DRS) analysis indicated that Ce doping extends the light absorption edge of FeOCl, enhancing visible light utilization. Additionally, PL spectra and I-T curve analyses demonstrate a marked reduction in the recombination rate of photogenerated charge carriers in FeOCl/Ce, which contributes to its improved performance in PS-mediated CAF degradation.

#### 2.2.2. Comparison of CAF Degradation in Different Systems

To investigate the effects of visible light irradiation, PS, and the photocatalyst on CAF degradation efficiency, controlled variable experiments were performed. Figure 4b shows the CAF degradation efficiency of FeOCl/Ce under different systems. It is evident that FeOCl/Ce alone exhibits poor dark adsorption of CAF, likely attributed to its weak adsorption capacity—consistent with the measured BET specific surface area of only 2.0 m^2^/g, which limits the number of available effective adsorption sites. Additionally, the redox potential of PS is merely 2.01 V, significantly lower than that of SO_4_^•−^ (*E_0_* = 2.5–3.1 V) [15,31], indicating its limited oxidative capacity. Furthermore, visible light alone is ineffective at activating PS, resulting in a degradation rate of only 11.6% for the Vis/PS system. The Vis + FeOCl/Ce system achieved a degradation rate of 32.6% for CAF, confirming that FeOCl/Ce can respond to visible light and exhibits inherent photocatalytic activity. The FeOCl/Ce + PS system, which relies on Fenton-like reactions between FeOCl/Ce and PS, degraded 44.1% of CAF within 1 min, with negligible further degradation thereafter. This phenomenon may be attributed to the relatively high dosage of FeOCl/Ce (0.5 g/L), which consumes most of the PS within this period. Notably, the combination of visible light irradiation and sulfate radical-based advanced oxidation processes yielded the highest CAF degradation rate of 91.2%. The results clearly demonstrated that visible light played a pivotal role in optimizing the photocatalytic performance of the FeOCl/Ce + PS system, primarily by facilitating photogenerated charge separation and enhancing the generation of reactive oxygen species.

### 2.3. Effects of Important Parameters on the Degradation Efficiency

#### 2.3.1. Effect of FeOCl/Ce Dosage

**Figure 5:**
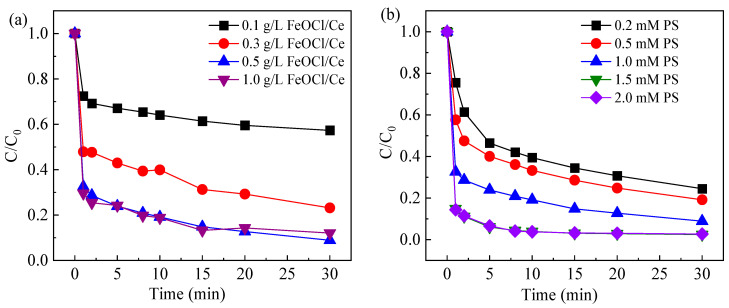
(**a**) Degradation of CAF under different FeOCl/Ce dosage and (**b**) degradation of CAF under different PS concentration. Experimental conditions: [CAF] = 10 μM, [PS] = 1 mM or [catalyst] = 0.5 g/L, [initial pH] = 5.06.

When the initial concentration of CAF was 10 μM, the PS concentration was 1 mM, and the initial pH of the system was 5.06, the effect of varying FeOCl/Ce dosage on CAF degradation efficiency under visible light irradiation was investigated, as shown in Figure 5a. As the FeOCl/Ce dosage increased from 0.1 g/L to 0.5 g/L, the degradation rate of CAF within 30 min increased from 42.7% to 91.1%. This improvement is presumably attributed to the higher catalyst dosage generating more photogenerated electrons and holes, while the increased level of soluble iron species provides more favorable conditions for SO_4_^•−^ generation. However, when the amount of FeOCl/Ce was increased to 1 g/L, the degradation rate of CAF (87.9%) did not improve but rather decreased, indicating that the degradation of CAF was inhibited. This may be attributed to the self-quenching of excessive free radicals (Equations (1)–(3)) [32,33,34,35]. Additionally, an excess of the photocatalyst can make the system turbid, affecting the light penetration and thus reducing the utilization of visible light. The SO_4_^•−^ in the system can also be directly scavenged by Fe^2+^ released from FeOCl/Ce (Equation (4)) [21]. Therefore, the optimal catalyst dosage selected for this study was 0.5 g/L.

SO_4_^•−^ + SO_4_^•−^ → S_2_O_8_^2−^ (1)

SO_4_^•−^ + ^•^OH → HSO_5_^−^ (2)

^•^OH + ^•^OH → H_2_O_2_ (3)

SO_4_^•−^ + Fe(II) → SO_4_^2−^ + Fe(III) (4)

The influence of PS concentration on the degradation efficiency of CAF under visible light was evaluated, with the results shown in Figure 5b. Increasing PS concentration from 0.2 mM to 1.5 mM enhanced CAF degradation from 75.5% to 97.6%, indicating that higher PS availability promotes the formation of reactive species within the FeOCl/Ce-enabled system, provided that sufficient FeOCl/Ce is present. It is noteworthy that when the PS concentration was further increased from 1.5 mM to 2 mM, the degradation rate of CAF approached nearly 100%, and there was no significant increase in degradation rate. This is because an excessive number of PS can deplete SO_4_^•−^ (as shown in Equation (5)) [36], and a surplus of free radicals may lead to self-quenching effects (as shown in Equation (1)). Considering both cost and the degradation efficiency of CAF, a PS concentration of 1 mM was selected as optimal for subsequent experiments.

SO_4_^•−^ + S_2_O_8_^2−^ → SO_4_^2−^ + S_2_O_8_^•−^ (5)

#### 2.3.2. Effect of Initial Solution pH

**Figure 6:**
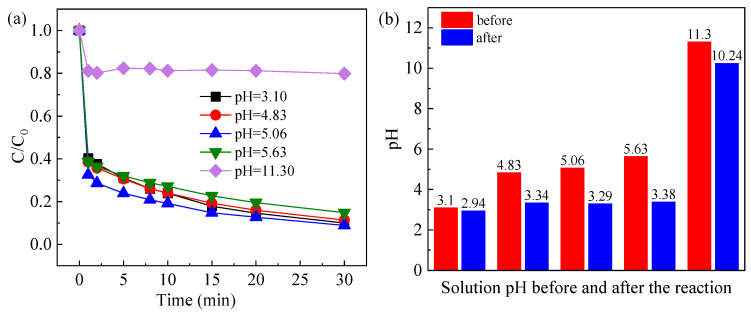
(**a**) Degradation of CAF under different pH levels and (**b**) pH of the system before and after the reaction. Experimental conditions: [CAF] = 10 μM, [PS] = 1 mM, [Catalyst] = 0.5 g/L.

When the initial concentration of CAF was 10 μM, FeOCl/Ce was added at 0.5 g/L, and the PS concentration was 1 mM, the influence of initial solution pH on the degradation efficiency of CAF under visible light irradiation was evaluated (Figure 6a). The results show that FeOCl/Ce exhibited excellent catalytic activity over an initial pH range of 3.10–5.63. The transient pH of the system after adding PS was 5.06 (i.e., unadjusted). As the initial pH increased from 3.10 to 5.63, the CAF degradation efficiency remained largely constant. However, when the initial pH increased to 10.30, the CAF degradation rate dropped to only 20.2%. The observed phenomenon may be attributed to the greater solubility of Fe ions at low pH, which facilitates the activation of PS. Figure 6b depicts the system pH before and after the reaction. From the figure, it can be inferred that the pH decreased as the reaction progressed, and when the initial pH ranged from 3.10 to 5.63, the post-reaction pH stabilized at 3.2 ± 0.2. This stabilization can be ascribed to the precipitation of iron hydroxide, formed by Fe species that precipitate during the reaction, which gradually stabilizes the solution pH. Given considerations of operational ease and the CAF degradation performance, pH adjustment was not required in subsequent experiments.

#### 2.3.3. Effect of Common Anions

**Figure 7:**
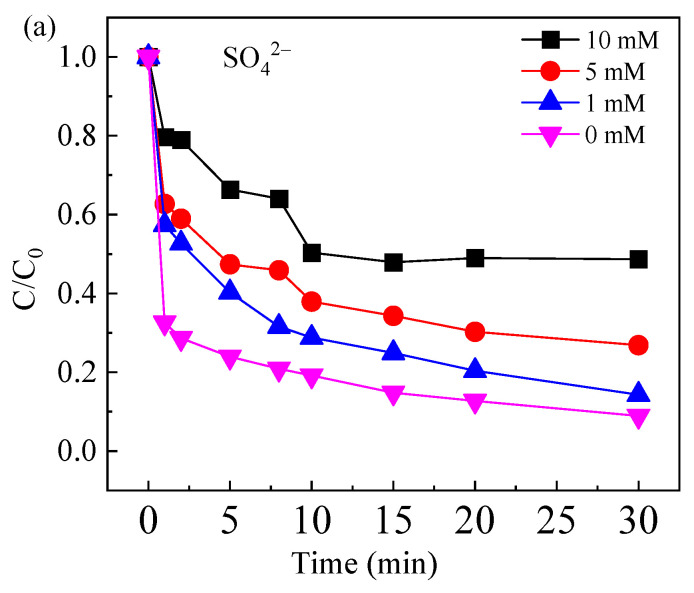

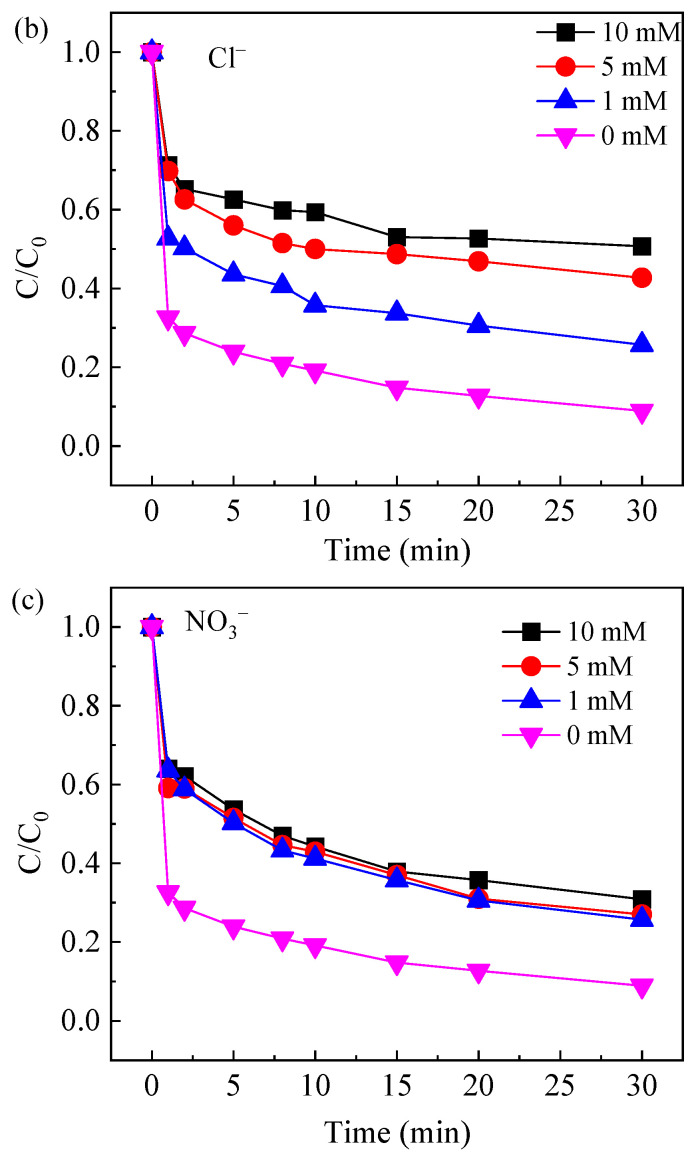
Effect of inorganic anions in water on the degradation of CAF: (**a**) SO_4_^2−^, (**b**) Cl^−^, and (**c**) NO_3_^−^. Experimental conditions: [CAF] = 10 μM, [PS] = 1 mM, [catalyst] = 0.5 g/L, [initial pH] = 5.06.

When the initial concentration of CAF was 10 μM, the FeOCl/Ce dosage was 0.5 g/L, the PS concentration was 1 mM, and the initial pH of the system was 5.06, the effects of common inorganic anions (SO_4_^2−^, Cl^−^, and NO_3_^−^) on the degradation efficiency of CAF were investigated, as illustrated in Figure 7. The results indicate that SO_4_^2−^, Cl^−^, and NO_3_^−^ had varying degrees of inhibitory effects on the degradation of CAF. As the concentrations of SO_4_^2−^ and Cl^−^ increased, the degradation of CAF decreased. Specifically, from Figure 7a, it can be observed that increasing SO_4_^2−^ concentration from 1 mM to 10 mM reduced the degradation rate of CAF from 85.8% to 51.4%. This behavior can be attributed to SO_4_^2−^ being strongly adsorbed onto or coordinated with Fe(III)/Fe(II) sites on the photocatalyst surface and thereby occupying active sites or preventing PS from approaching and being activated via electron transfer. The higher SO_4_^2−^ load intensifies these complexation reactions, reducing the free sites of Fe(III) and Fe(II) due to coordination. Besides, the presence of SO_4_^2−^ also lowers the redox potential of SO_4_^•−^/SO_4_^2−^, thereby affecting the catalytic efficiency of PS activation [37].

As illustrated in Figure 7b, an increase in chloride ion (Cl^−^) concentration from 1 mM to 10 mM corresponded to a decrease in the degradation rate of CAF from 74.4% to 49.4%; this phenomenon arises because Cl^−^ can react with SO_4_^•−^ to generate less reactive free radicals, specifically chlorine radicals (Cl^•^, 2.41 V) and dichloride radicals (Cl_2_^•−^, 2.09 V), as shown in Equations (6) and (7) [38]. As depicted in Figure 7c, the introduction of nitrate ions resulted in a reduced CAF degradation rate. This is presumably due to the formation of a passivation layer on the surface of the FeOCl/Ce composite; this passivation layer impairs the generation of photogenerated electrons and holes, thereby exerting a negative effect on the activation of PS. Notably, when the concentration of NO_3_^−^ increased from 1 to 10 mM, the CAF degradation rate remained relatively stable. This stability can be attributed to two key factors: Firstly, NO_3_^−^ reacts with SO_4_^•−^ to form nitrate radicals (NO_3_^•^) with a comparatively lower reactivity (Equation (8)) [31,37,39], and these NO_3_^•^ radicals exhibit a low secondary reaction rate constant. Secondly, once the passivation layer is formed, further increases in NO_3_^−^ concentration do not promote the progression of passivation reactions.

SO_4_^•−^ + Cl^−^ ↔ SO_4_^2−^ + Cl^•^ *k*_f_ = 3.2 × 10^8^ M^−1^ s^−1^, *k*_b_ = 2.1 × 10^8^ M^−1^ s^−1^ (6)

Cl^•^ + Cl^−^↔ Cl_2_^•−^ *k*_f_ = 6.5 × 10^9^ M^−1^ s^−1^, *k*_b_ = 1.1 × 10^5^ M^−1^ s^−1^ (7)

SO_4_^•−^ + NO_3_^−^ →SO_4_^2−^ + NO_3_^•^, *k* = 2.1 × 10^6^ M^−1^ s^−1^ (8)

### 2.4. Evaluation of the Stability of FeOCl/Ce Catalyst

**Figure 8:**
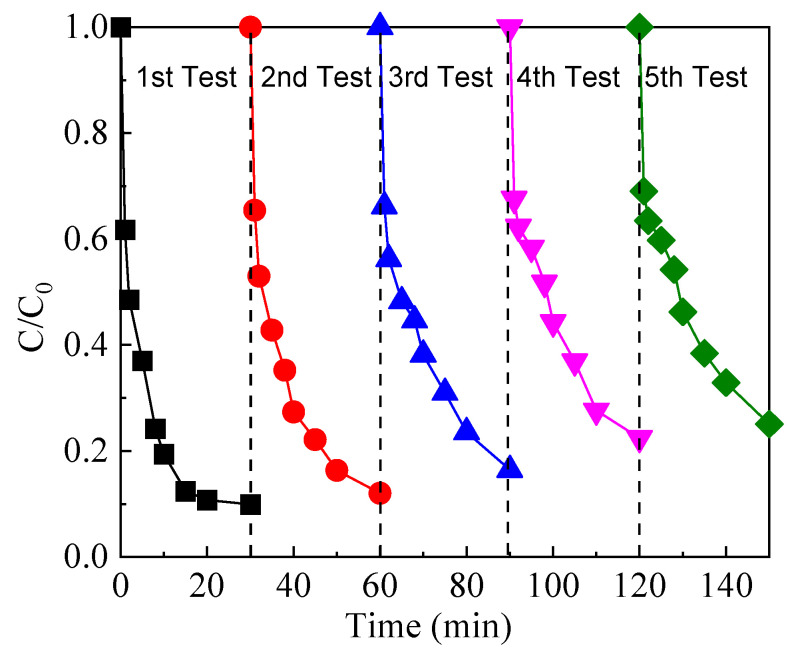
Reusability of FeOCl/Ce. Experimental conditions: [CAF] = 10 μM, [PS] = 1 mM, [catalyst] = 0.5 g/L, [initial pH] = 5.06.

The sustained catalytic performance of a catalyst is a crucial attribute for practical applications. Accordingly, the reusability of the prepared catalyst was evaluated over five consecutive cycles. As shown in Figure 8, the recovered photocatalyst maintained favorable performance, with the CAF removal efficiency exhibiting a slight, gradual decline from 90.1% to 63% after five runs. The attenuation of its catalytic activity may be attributed to the continuous leaching of Fe ions. Furthermore, the adsorption of CAF or its degradation intermediates onto the surface of the FeOCl/Ce composite could hinder the exposure of its active sites and reduce accessibility, thereby resulting in a reduced catalytic efficiency.

### 2.5. Degradation Mechanisms

**Figure 9:**
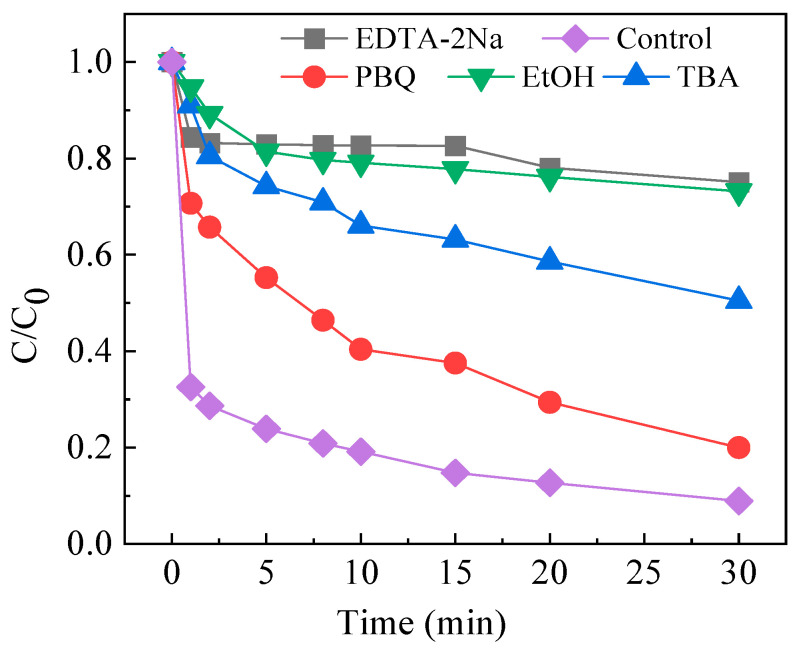
Effect of different scavengers on the degradation of CAF by FeOCl/Ce under visible light irradiation. Experimental conditions: [CAF] = 10 μM, [PS] = 1 mM, [catalyst] = 0.5 g/L, [initial pH] = 5.06.

To identify the reactive species present in the system, free radical quenching experiments were conducted by introducing ethylenediaminetetraacetic acid disodium salt (EDTA-2Na), p-benzoquinone (PBQ), ethanol (EtOH), and tert-butyl alcohol (TBA) as specific scavengers for holes (h^+^), O_2_^•−^, SO_4_^•−^, and ^•^OH, respectively [40,41,42]. The experimental results are shown in Figure 9. Compared with the blank control (i.e., no inhibitor), the degradation of CAF was retarded to varying degrees upon the addition of each inhibitor, indicating the involvement of multiple reactive species. The introduction of EDTA-2Na reduced the degradation rate of CAF to 25.0%, indicating a significant role of holes in the system. After adding PBQ to the system, the degradation rate of CAF decreased to 80.1%, confirming the participation of O_2_^•−^. It is known that EtOH containing α-hydrogen rapidly scavenges both SO_4_*^•^*^−^ and ^•^OH due to its high reactivity toward these two radicals with rate constants of (1.6–7.7) × 10^7^ M^−1^·s^−1^ for SO_4_*^•^*^−^ and (1.2–2.8) × 10^9^ M^−1^·s^−1^ for ^•^OH, respectively [42]. In contrast, TBA, which lacks α-hydrogen, reacts with ^•^OH at (3.8–7.6) × 10^8^ M^−1^·s^−1^, i.e., approximately three orders of magnitude faster than that with SO_4_*^•^*^−^, which reacts at (4.0–9.1) ×10^5^ M^−1^·s^−1^ [43]. Consequently, TBA is commonly employed as a selective scavenger for ^•^OH. By comparing degradation efficiencies in the presence of distinct alcohol scavengers (i.e., EtOH and TBA), the respective contributions of SO_4_*^•^*^−^ and ^•^OH can be distinguished. After introducing 10 mM TBA, the degradation rate of CAF dropped from 91.2% to 49.5%, indicating the dominating involvement of ^•^OH in the system. Following the addition of EtOH, the degradation rate of CAF further declined to 26.8%. The inhibitory difference between EtOH and TBA suggested that SO_4_*^•^*^−^ radical species also contributed to the decay of CAF in the system. It is worth noting that the degrees of inhibition follow the order EDTA-2Na > EtOH > TBA > BQ, indicating that h^+^ plays a significant role in the photocatalytic degradation, while ^•^OH and SO_4_*^•^*^−^ are the other major reactive species driving the reaction.

**Figure 10:**
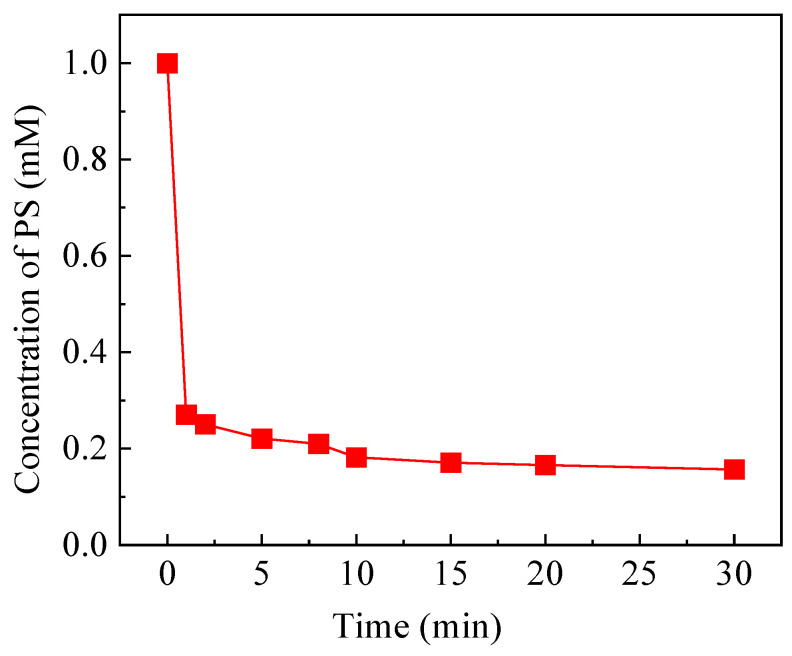
The temporal evolution of PS concentration in the system as a function of reaction time. Experimental conditions: [CAF] = 10 μM, [PS] = 1 mM, [catalyst] = 0.5 g/L, [initial pH] = 5.06.

To further elucidate the mechanism of FeOCl/Ce catalyzing the decomposition of PS under visible light for the degradation of CAF, the temporal evolution of PS concentration in the system was monitored. Figure 10 presents the variation of PS concentration as a function of reaction time. A rapid initial decline in PS concentration within the first minute of the reaction was observed, decreasing from the initial concentration of 1 to 0.27 mM, after which the rate of PS consumption approaches a retarding stage. The observed temporal evolution of PS concentration aligns with the degradation trends of CAF under different systems, as shown in Figure 4b. The FeOCl/Ce + PS system achieved 44.1% CAF degradation within the first minute, with negligible further degradation thereafter. This limited performance may be attributed to the relatively high dosage of FeOCl/Ce (0.5 g/L) relative to PS, which led to a rapid consumption of most PS within the initial minute. In contrast, the Vis + FeOCl/Ce + PS system attained 67.5% of CAF degradation in the first minute, after which the reaction rate markedly decelerated. Therefore, the changes in PS concentration over the reaction time in the system are consistent with the results discussed above.

**Figure 11:**
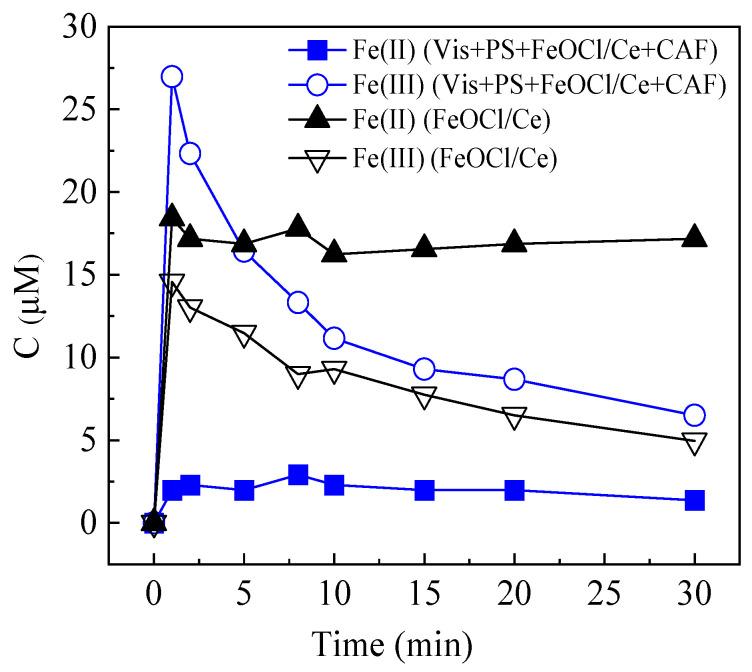
Concentration of soluble iron in the system. Experimental conditions: [CAF] = 10 μM, [PS] = 1 mM, [catalyst] = 0.5 g/L, [initial pH] = 5.06.

The redox transformations of iron species within the system are crucial for understanding the photocatalytic activity of FeOCl/Ce and its efficiency in catalyzing PS decomposition. Under visible light irradiation, the FeOCl/Ce + PS system accelerates the Fe(II)/Fe(III) redox cycle by trapping photoexcited electrons, which facilitates the separation of electrons and h^+^ and inhibits their recombination, consistent with the mechanism reported by Qu et al. for FeOCl-activated peroxylmonosulfate under visible light [14]. Specifically, Ce doping broadens the optical absorption edge of FeOCl by introducing new energy levels within its bandgap via the 4f electronic configuration of Ce, thus extending its visible light response range. This allows FeOCl/Ce to capture more photons under visible light, generating a greater number of photogenerated electrons and holes compared to pure FeOCl. Furthermore, Ce^3+^/Ce^4+^ ions act as electron-trapping wells. During photocatalysis, photogenerated electrons from the conduction band of FeOCl can be captured by Ce^4+^ to form Ce^3+^, while holes remain in the valence band. This valence cycling of Ce^3+^/Ce^4+^ further prolongs the separation lifetime of electron-hole pairs, effectively reducing their recombination rate. As evidenced by PL spectra, FeOCl/Ce exhibits lower PL intensity than pure FeOCl, directly confirming the suppressed charge recombination. Subsequently, the accumulated electrons stored in Ce^3+^ and the conduction band of FeOCl efficiently activate PS to generate sulfate and hydroxyl radicals. Besides, the iron ions released may further assist in boosting the process performance. To elucidate the role of soluble iron ions in this system, experiments were conducted with an initial CAF concentration of 10 μM, FeOCl/Ce dosage of 0.5 g/L, and PS concentration of 1 mM, while changes in the concentration of soluble iron were monitored throughout the process. As shown in Figure 11, FeOCl/Ce composite releases Fe(II) into the aqueous solution. Within the first minute of the reaction, the concentration of Fe(II) increases rapidly; meanwhile, a portion of the released Fe(II) is oxidized to Fe(III). After this initial stage, the increase in Fe(II) concentration slows down and gradually approaches an apparent equilibrium state. In contrast, the concentration of Fe(III) decreases over time. This decline in Fe(III) concentration is attributed to the precipitation of iron hydroxide on the surface of FeOCl/Ce catalyst, which acts as a barrier and thereby inhibits the further release of Fe(II) into the solution.

In the Vis + PS + FeOCl/Ce + CAF system, the FeOCl/Ce catalyst rapidly releases Fe(II) within the first minute of the reaction. Subsequently, two key reactions involving Fe(II) occur: first, Fe(II) reacts with persulfate (PS) to generate Fe(III) and SO_4_*^•^*^−^; secondly, Fe(II) can further react with SO_4_*^•^*^−^ to form additional Fe(III). As a result, the concentration of Fe(III) in the system remains relatively high after one minute of the reaction. Thereafter, the Fe(III) concentration exhibits a downward trend, which can be attributed to two factors: the formation of iron hydroxides and the capacity of Fe(III) to capture photogenerated electrons and participate in subsequent reactions. Notably, the ability of Fe(III) to trap photogenerated electrons plays a critical role, which can effectively facilitate the separation of photogenerated electron–hole pairs. This enhanced separation of charge carriers, in turn, improves the photocatalytic activity of the FeOCl/Ce catalyst.

## 3. Materials and Methods

### 3.1. Chemicals

Caffeine (C_8_H_10_N_4_O_2_, 99%), ferric chloride (FeCl_3_, >99.9%), Cerium Chloride (CeCl_3_, >99.9%), potassium persulfate (K_2_S_2_O_8_, ≥99.0%), benzoquinone (C_6_H_4_O_2_ 98%), sodium chloride (NaCl, ≥99.5%), and sodium hydroxide (NaOH, ≥98.0%), ethanol (EtOH), and *tert*-butyl alcohol (TBA) were purchased from Sigma-Aldrich Inc. (Burlington, MA, USA). All chemicals are of analytic reagent-grade, and all solvents are of high-performance liquid chromatography (HPLC)-grade and used as received without further purification. Distilled–deionized water with a resistivity of 18.25 MΩ cm^−1^ generated by a Cascada^TM^ BIO water purification system (Pall Corporation, Redruth, UK) was used for the preparation of all solutions. The initial pH of the solutions was adjusted using sulfuric acid and/or sodium hydroxide.

### 3.2. Catalyst Preparation

Synthesis of FeOCl. Pure phase FeOCl was prepared using a partial pyrolysis method modified from a previous study [14]. An appropriate amount of anhydrous FeCl_3_ was ground in an agate mortar for 5 min and then transferred to a crucible. The crucible was placed in a furnace at 250 °C for 1 h, then cooled to room temperature. After removing it, the material was thoroughly ground and washed with anhydrous ethanol after centrifuging for 5 min, repeating this process 4–5 times to remove any unreacted FeCl_3_. Finally, the sample was dried in a vacuum oven at 60 °C for 2 h and ground again to obtain the final product.

Synthesis of FeOCl/Ce. Anhydrous FeCl_3_ and CeCl_3_·7H_2_O powder were mixed in a crucible at a molar ratio of 10:1. After adding 2 mL of ultrapure water to the crucible and stirring thoroughly, the mixture was placed in a vacuum oven at 60 °C to dry for 13 h, forming a precursor. The precursor was then placed in a furnace at 250 °C for 1 h, cooled to room temperature, removed, and thoroughly ground. The washing process with anhydrous ethanol was carried out using centrifugation for 5 min, repeating 4–5 times to remove any unreacted FeCl_3_. Finally, the sample was dried in a vacuum oven at 60 °C for 2 h and ground to obtain the final product.

### 3.3. Characterization

The crystallinity, purity, and phase composition of the photocatalysts were detected using a Bruker D8 Advance X-ray diffraction (XRD) (Bruker-AXS, Karlsruhe, Germany) device. An MLA650F scanning electron microscope (FEI, Ocala, FL, USA) coupled with a Bruker XFlash 6I30 energy dispersive spectroscopy (SEM-EDS) (Bruker-AXS, Karlsruhe, Germany) instrument was utilized to analyze the surface morphology, elemental distribution, and composition of the samples. X-ray photoelectron spectroscopy (XPS, AXIS ULTRA, Tokyo, Japan) was employed to detect the valence states of various elements in the photocatalysts. The BET surface area test was used to determine the pore size and specific surface area of the samples. A Bruker Tensor27 Fourier-transform infrared spectroscopy (FT-IR) (Bruker-AXS, Karlsruhe, Germany) was employed to analyze the types of functional groups, molecular structure, and chemical bonds in the photocatalyst. Ultraviolet-visible diffuse reflectance spectroscopy (DRS, Lambd 1050, Perkin-Elmer, Alameda, CA, USA) was performed to determine the light absorption edge of the samples in the ultraviolet to visible spectrum, thereby inferring the light absorption properties of the photocatalyst and estimating its light absorption threshold and bandgap width. A Edinburgh FLS980 Photoluminescence spectroscopy (PL) (Edinburgh Instruments, Scotland, UK) was used to compare the recombination rates of photogenerated electrons and holes in the photocatalyst. The current–time (I-T) curves were measured by an electrochemical workstation (CHI660D, CH Instruments Inc., Shanghai, China) to compare the photoelectric performance of the photocatalyst.

### 3.4. Photocatalytic Degradation Experiment

**Figure 12:**
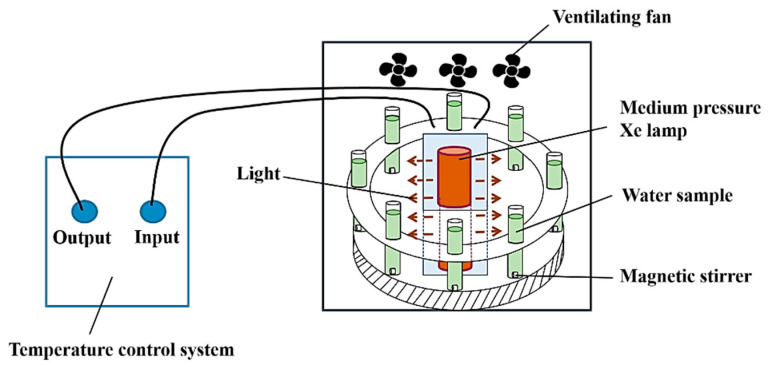
Schematic diagram of the photochemical reactor.

Degradation experiments were conducted in a multi-channel photochemical reaction apparatus, with a schematic diagram of the experimental setup shown in Figure 12. The light source used in the experiment was a 500 W Xe lamp, and the reaction solution was placed in fixed channels. The photochemical reaction apparatus was equipped with an exhaust fan and a low-temperature cooling liquid circulation pump to provide a constant temperature environment for the experimental system; a magnetic stirring system was used to ensure a uniform reaction.

Before the experiment, the low-temperature cooling liquid circulation pump was adjusted to 15 °C, the exhaust fan was turned on, and the Xe lamp was turned on 5 min in advance to ensure that the light source was stable during the experiment. The initial concentration of CAF was 10 μM with a total reaction volume of 50 mL in the test tube. After adding a quantified amount of photocatalyst and PS, the test tube was quickly placed in the fixed channel to initiate the photochemical reaction. Samples were taken at fixed time, filtered through a 0.22 μm filter membrane, and 1.2 mL of the filtrate was quenched with 0.1 mL of methanol (MeOH). High-performance liquid chromatography (HPLC) was used to determine the concentration of the target contaminant in the solution. The pH was adjusted using H_2_SO_4_ at pH_3_ and NaOH at pH_12_.

The influence of different anions on the experiment was conducted by adding varying concentrations of Cl^−^, SO_4_^2−^, and NO_3_^−^. The recycle experiments involve washing the samples after the photocatalytic activity tests with deionized water three times for recovery. Since sample loss may occur during the recovery process, parallel experiments were performed for each recycle experiment to recover the samples, following the same steps under identical conditions. The radical scavenging experiments involve adding 10 mM of disodium ethylenediaminetetraacetate (EDTA-2Na), ethanol (EtOH), tert-butanol (TBA), and 1 mM of p-benzoquinone (PBQ) to the system before the reaction starts, with all other steps remaining the same.

### 3.5. Analytical Testing Methods

#### 3.5.1. Determination of CAF

The concentration of CAF before and after the reaction was quantified using high-performance liquid chromatography (HPLC) (Thermo Fisher Scientific Inc., Waltham, MA, USA). The chromatographic conditions were as follows: the detection wavelength was set at 273 nm; the mobile phase consisted of methanol and formic acid (1‰) in a 50%:50% (*v*:*v*) ratio, with a flow rate of 0.2 mL/min; the separation column used was a Thermo Fisher Accucore™ (Waltham, MA, USA) C18 (150 mm × 2.1 mm ID) HPLC column; the column temperature was maintained at 30 °C; the injection volume for CAF was 5 μL; and the retention time for CAF was 1.8 min.

#### 3.5.2. Determination of PS Concentration

The concentration of PS was quantified by the iodometric ultraviolet spectrophotometric method [44]. Specifically, PS can oxidize iodide to iodine under neutral pH conditions, producing a yellow-colored aqueous solution whose absorbance is measured at 352 nm using a UV-Vis spectrophotometer (Beifen-Ruili Analytical Instrument (Group) Co., Ltd., Beijing, China).

## 4. Conclusions

In this study, FeOCl/Ce composite photocatalysts were successfully synthesized via a partial pyrolysis approach, and the physicochemical properties of the as-prepared photocatalysts were systematically characterized using multiple advanced analytical techniques. Experimental results demonstrate that Ce doping effectively reduces the bandgap energy of the photocatalyst, enhances its light absorption capacity, and significantly suppresses the recombination rate of photogenerated electron–hole pairs, thereby improving its photocatalytic performance under visible light. Both pure FeOCl and FeOCl/Ce achieved over 90% CAF removal within 30 min under visible light irradiation; however, FeOCl/Ce shows a significantly higher degradation kinetic rate constant. Ce doping broadens the optical absorption edge of FeOCl and increases the utilization efficiency of visible light. PL spectra and I-T measurements indicate that FeOCl/Ce has a markedly reduced recombination rate of photogenerated carriers, thus improving its catalytic activation of PS for the degradation of CAF in aqueous solution. When the initial concentration of CAF is 10 μM, the dosage of FeOCl/Ce is 0.5 g/L, the PS concentration is 1 mM, and the initial pH is 5.06, FeOCl/Ce can degrade 91.2% of CAF under visible light in 30 min. Within the pH range of 3.10–11.3, an acidic pH environment is more favorable for the degradation of CAF. The presence of SO_4_^2−^, Cl^−^, and NO_3_^−^ exerts inhibitory effects on CAF degradation to varying degrees, possibly due to their competitive adsorption on the photocatalyst surface or quenching of reactive radical species. Cyclic stability tests show that FeOCl/Ce exhibited favorable catalytic performance and reusability in multiple runs. In this system, ^•^OH and holes are the dominant reactive species responsible for CAF degradation, while PS mainly functions as an electron acceptor to suppress electron-hole pair recombination, thus enhancing photocatalytic activity.

Overall, the FeOCl/Ce-based heterogeneous photocatalytic system exhibits excellent performance in CAF degradation, with advantages of high efficiency, good stability, and visible light responsiveness. These characteristics confirm that FeOCl/Ce has great potential as a promising catalyst for the removal of caffeine and other emerging organic pollutants from aqueous environments.

## Figures and Tables

**Table 1:** Analysis of surface element content of FeOCl/Ce.

Element	Mass Norm (%)	Atom (%)	Abs. Error (%)1 Sigma
C	14.88	28.48	0.98
O	9.30	13.36	0.59
Al	60.22	51.29	1.58
Cl	1.95	1.26	0.06
Fe	13.60	5.60	0.22
Ce	0.04	0.01	0.00
Sum	100.00	100.00	

## Data Availability

Data available on request from the authors.

## References

[1] Hawash H.B., Moneer A.A., Galhoum A.A., Elgarahy A.M., Mohamed W.A.A., Samy M., El-Seedi H.R., Gaballah M.S., Mubarak M.F., Attia N.F. (2023). Occurrence and spatial distribution of pharmaceuticals and personal care products (PPCPs) in the aquatic environment, their characteristics, and adopted legislations. J. Water Process Eng..

[2] Liu N., Jin X., Johnson A.C., Zhou S., Liu Y., Hou L., Meng F., Wu F. (2025). Pharmaceutical and Personal Care Products (PPCPs) in Global Surface Waters: Risk and Drivers. Environ. Sci. Technol..

[3] Wang H., Xi H., Xu L., Jin M., Zhao W., Liu H. (2021). Ecotoxicological effects, environmental fate and risks of pharmaceutical and personal care products in the water environment: A review. Sci. Total Environ..

[4] Kumar M., Sridharan S., Sawarkar A.D., Shakeel A., Anerao P., Mannina G., Sharma P., Pandey A. (2023). Current research trends on emerging contaminants pharmaceutical and personal care products (PPCPs): A comprehensive review. Sci. Total Environ..

[5] Yang Y., Zhang X., Jiang J., Han J., Li W., Li X., Leung K.M.Y., Snyder S.A., Alvarez P.J.J. (2022). Which Micropollutants in Water Environments Deserve More Attention Globally?. Environ. Sci. Technol..

[6] Yu Y., Wang Z., Yao B., Zhou Y. (2024). Occurrence, bioaccumulation, fate, and risk assessment of emerging pollutants in aquatic environments: A review. Sci. Total Environ..

[7] Yan R., Han J., Shen G., Hao Z., Han Y., Xiong W., Liang B., Gao S., Yang M., Sun Y. (2025). The threat of PPCPs from WWTP and solutions of advanced reduction coupled treatment processes with pilot-scale. J. Hazard. Mater..

[8] Lorentz B., Rauhauser M., Krantz R.T., Snow D.D., Kelly J.J. (2025). Treated wastewater effluent increases pharmaceutical concentrations and alters benthic microbial communities in streams. Front. Microbiol..

[9] Zhang Q., Zheng D., Bai B., Ma Z., Zong S. (2024). Insight into antibiotic removal by advanced oxidation processes (AOPs): Performance, mechanism, degradation pathways, and ecotoxicity assessment. Chem. Eng. J..

[10] Tabatabaei M., Cho D.-W., Fahad S., Jeong D.-W., Hwang J.-H. (2025). Photocatalytic innovations in PFAS removal: Emerging trends and advances. Sci. Total Environ..

[11] Bhapkar A.R., Bhame S. (2024). A review on ZnO and its modifications for photocatalytic degradation of prominent textile effluents: Synthesis, mechanisms, and future directions. J. Environ. Chem. Eng..

[12] Akerdi A.G., Bahrami S.H. (2019). Application of heterogeneous nano-semiconductors for photocatalytic advanced oxidation of organic compounds: A review. J. Environ. Chem. Eng..

[13] Yang X.-J., Xu X.-M., Xu J., Han Y.-F. (2013). Iron Oxychloride (FeOCl): An Efficient Fenton-Like Catalyst for Producing Hydroxyl Radicals in Degradation of Organic Contaminants. J. Am. Chem. Soc..

[14] Qu S., Li C., Sun X., Wang J., Luo H., Wang S., Ta J., Li D. (2019). Enhancement of peroxymonosulfate activation and utilization efficiency via iron oxychloride nanosheets in visible light. Sep. Purif. Technol..

[15] Zhao X., Zhang Z. (2023). FeOCl in Advanced Oxidization Processes for Water Purification: A Critical Review. Curr. Pollut. Rep..

[16] Jiang S., Zheng H., Sun X., Zhu M., Zhou Y., Wang D., Zhang D., Zhang L. (2022). New and highly efficient Ultra-thin g-C_3_N_4_/FeOCl nanocomposites as photo-Fenton catalysts for pollutants degradation and antibacterial effect under visible light. Chemosphere.

[17] Keskin O.Y., Dalmis R., Birlik I., Azem N.F.A. (2020). Comparison of the effect of non-metal and rare-earth element doping on structural and optical properties of CuO/TiO_2_ one-dimensional photonic crystals. J. Alloys Compd..

[18] Jiang S., Liu Y., Xu J. (2021). Rare earth oxynitrides: Promising visible-light-driven photocatalysts for water splitting. Mater. Adv..

[19] Seles P., Vengust D., Radosevic T., Kocijan M., Einfalt L., Kurtjak M., Shvalya V., Knaflic T., Bernik S., Omerzu A. (2024). Altering defect population during the solvothermal growth of ZnO nanorods for photocatalytic applications. Ceram. Int..

[20] Sabri M., Habibi-Yangjeh A., Chand H., Krishnan V. (2020). Activation of persulfate by novel TiO_2_/FeOCl photocatalyst under visible light: Facile synthesis and high photocatalytic performance. Separ. Purif. Technol..

[21] Chen M., Xu H., Zhang X., Li D., Xia D. (2018). Mechanism of heterogeneous activation of persulfate with FeOCl under visible light irradiation. J. Mater. Res..

[22] Geng J., Zhu G., Yang Y., Zhang X., Wang D., Wang J., Zhu Y. (2024). Enhanced antibiotic mineralization and detoxification through photoregeneration of FeOCl surface active site. Chem. Eng. J..

[23] Chen R., Jiang H., Li Y.-Y. (2018). Caffeine degradation by methanogenesis: Efficiency in anaerobic membrane bioreactor and analysis of kinetic behavior. Chem. Eng. J..

[24] Shi X., Cui C., Zhang L., Zhang J., Liu G. (2019). FeOCl/Ln (Ln = La or Y): Efficient photo-Fenton catalysts for ibuprofen degradation. New J. Chem..

[25] Chen M., Xu H., Wang Q., Li D., Xia D. (2018). Activation mechanism of sodium percarbonate by FeOCl under visible-light-enhanced catalytic oxidation. Chem. Phys. Lett..

[26] Baer D.R., Engelhard M.H., Johnson G.E., Laskin J., Lai J., Mueller K., Munusamy P., Thevuthasan S., Wang H., Washton N. (2013). Surface characterization of nanomaterials and nanoparticles: Important needs and challenging opportunities. J. Vac. Sci. Technol. A.

[27] Zhang J., Yang M., Lian Y., Zhong M., Sha J., Liu G., Zhao X., Liu S. (2019). Ce^3+^ self-doped CeO_x_/FeOCl: An efficient Fenton catalyst for phenol degradation under mild conditions. Dalton Trans..

[28] Hu M., Wang Y., Fan J., Li M., Rao Y. (2022). Activation of persulfate by nano zero-valent iron for degradation of emerging pollutant caffeine. Ind. Water Treat..

[29] Wu Y.H., Meng D.Q., Guo Q.B., Gao D., Wang L. (2022). Study on TiO_2_/g-C_3_N_4_ S-Scheme heterojunction photocatalyst for enhanced formaldehyde decomposition. Opt. Mater..

[30] Wang P., Zhou Q., Xia Y., Zhan S., Li Y. (2018). Understanding the charge separation and transfer in mesoporous carbonate doped phase-junction TiO_2_ nanotubes for photocatalytic hydrogen production. Appl. Catal. B Environ. Energy.

[31] Gao Y.J., Luo J.C., Song T.H., Yu X. (2021). Research progress on nano-Fe^0^/PS system for degradation of refractory organics in aqueous solution. J. Environ. Chem. Eng..

[32] Kim C., Ahn J.Y., Kim T.Y., Shin W.S., Hwang I. (2018). Activation of persulfate by nanosized zero-valent iron (NZVI): Mechanisms and transformation products of NZVI. Environ. Sci. Technol..

[33] Giannakis S., Lin K.-Y.A., Ghanbari F. (2021). A review of the recent advances on the treatment of industrial wastewaters by Sulfate Radical-based Advanced Oxidation Processes (SR-AOPs). Chem. Eng. J..

[34] Wang J., Wang S. (2020). Reactive species in advanced oxidation processes: Formation, identification and reaction mechanism. Chem. Eng. J..

[35] Devi P., Das U., Dalai A.K. (2016). In-situ chemical oxidation: Principle and applications of peroxide and persulfate treatments in wastewater systems. Sci. Total Environ..

[36] Lin C.-C., Wu M.-S. (2014). UV/S_2_O_8_^2−^ process for degrading polyvinyl alcohol in aqueous solutions. Chem. Eng. Process.-Process Intensif..

[37] Gao Y.-Q., Gao N.-Y., Wang W., Kang S.-F., Xu J.-H., Xiang H.-M., Yin D.-Q. (2018). Ultrasound-assisted heterogeneous activation of persulfate by nano zero-valent iron (nZVI) for the propranolol degradation in water. Ultrason. Sonochem..

[38] Farhat A., Keller J., Tait S., Radjenovic J. (2017). Assessment of the impact of chloride on the formation of chlorinated by-products in the presence and absence of electrochemically activated sulfate. Chem. Eng. J..

[39] Lin C.-C., Chen Y.-H. (2018). Feasibility of using nanoscale zero-valent iron and persulfate to degrade sulfamethazine in aqueous solutions. Separ. Purif. Technol..

[40] Trenczek-Zajac A., Synowiec M., Zakrzewska K., Zazakowny K., Kowalski K., Dziedzic A., Radecka M. (2022). Scavenger-Supported Photocatalytic Evidence of an Extended Type I Electronic Structure of the TiO_2_@Fe_2_O_3_ Interface. Acs Appl. Mater. Interfaces.

[41] Xu X., Zhang S., Wang Y., Lin Y., Guan Q., Chen C. (2023). Identifying the Role of Surface Hydroxyl on FeOCl in Bridging Electron Transfer toward Efficient Persulfate Activation. Environ. Sci. Technol..

[42] Liang C., Su H.-W. (2009). Identification of Sulfate and Hydroxyl Radicals in Thermally Activated Persulfate. Ind. Eng. Chem. Res..

[43] Zhu C., Zhu F., Dionysiou D.D., Zhou D., Fang G., Gao J. (2018). Contribution of alcohol radicals to contaminant degradation in quenching studies of persulfate activation process. Water Res..

[44] Liang C., Huang C.-F., Mohanty N., Kurakalva R.M. (2008). A rapid spectrophotometric determination of persulfate anion in ISCO. Chemosphere.

